# Flexible hybrid structure piezoelectric nanogenerator based on ZnO nanorod/PVDF nanofibers with improved output†

**DOI:** 10.1039/c8ra10315a

**Published:** 2019-04-01

**Authors:** Parisa Fakhri, Babak Amini, Roohollah Bagherzadeh, Mohammad Kashfi, Masoud Latifi, Neda Yavari, Soodeh Asadi Kani, Lingxue Kong

**Affiliations:** Textile Engineering Department, Amirkabir University of Technology, Textile Excellence & Research Centers Tehran Iran; Instrumentation Research Group, Niroo Research Institute (NRI) Tehran Iran bamini@nri.ac.ir; Institute for Advanced Textile Materials and Technologies, Textile Engineering Department, Amirkabir University of Technology Tehran Iran Bagherzadeh_r@aut.ac.ir; Mechanical Engineering Department, Ayatollah Boroujerdi University Boroujerd Iran; Institute for Frontier Materials, Deakin University, Geelong Campus VIC Australia

## Abstract

This study aimed to develop a novel hybrid piezoelectric structure based on poly(vinylidene difluoride) nanofibers (PVDF NFs) and zinc oxide nanorods (ZnO NRs) which eliminate the need for post poling treatment in such hybrid structures. Mechanism of electrical performance enhancement of the hybrid structure is also discussed in this paper. To study the effect of hybridization on piezoelectric performance, pristine ZnO NRs and pristine PVDF NF nanogenerators were also fabricated. The piezoelectric performance of these three nanogenerators was evaluated under periodic deformation at low frequency. The output power of the hybrid structure was found to be enhanced compared to pristine ZnO NRs and PVDF NFs nanogenerators. Such simple hybrid devices that do not need to complicated post poling treatment are more efficient than previous hybrid PVDF/ZnO nanogenerators for practical application. This improved piezoelectric nanogenerator is expected to enable various applications in the field of self-powered devices and wearable energy harvesting to harvest mechanical energy from the human activities.

## Abstract 2

A novel hybrid piezoelectric structure based on electrospun PVDF NFs and vertically grown ZnO nanorods is presented as a promising nanogenerator to convert mechanical movement more efficiently into electricity for practical applications.

## Graphical abstract

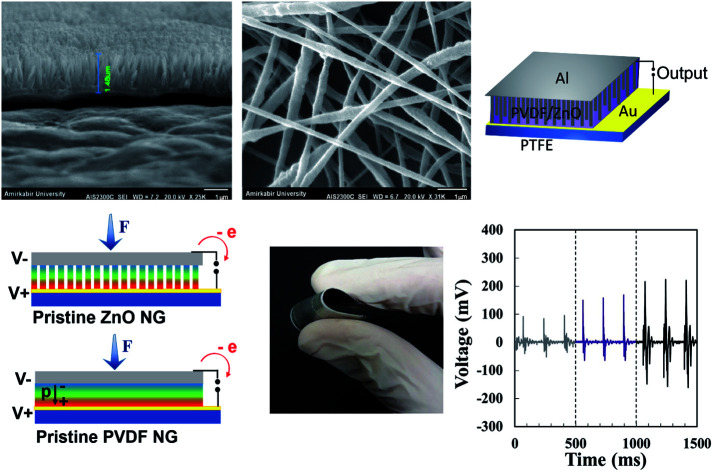

## Introduction

Piezoelectric nanogenerators (NGs) have attracted considerable attention in recent years due to their practical applications to convert mechanical energy from different sources to electrical energy.^1–7^ This technology can be used not only to harvest energy for application in self-powered systems, but also to detect physical motions as part of an active sensor.^8,9^ For such applications, zinc oxide (ZnO) nanostructures have been widely studied owing to their flexibility, biocompatibility and ease of growth.^10–12^ On the other hand, semi-crystalline PVDF is also well known as a piezoelectric polymer because of its electroactive properties, low cost, high mechanical strength, high chemical resistance, ease of processing and flexibility.^13–18^

Owing to the unique properties of ZnO and PVDF, to date many efforts have been made to improve the piezoelectric performance of nanogenerators based on these two piezoelectric materials. The hybridization of ZnO NWs with PVDF has been introduced in some research as a useful method to enhance piezoelectric performance and improve nanogenerator applications. Lee *et al.*^19^ by hybridization of ZnO NWs and PVDF, demonstrated a power source for a wearable electronic capable of scavenging energy from human activity. They stated that the PVDF acts as the piezoelectric ensemble with the ZnO NWs and a protective material to extend durability under deformation. Li *et al.*,^20^ enhanced the β phase and the stability of the PVDF film by *in situ* growth of ZnO nanowires. Lee *et al.*^21^ developed a highly sensitive and multifunctional tactile sensor using ZnO/PVDF thin film for pressure and temperature monitoring. They suggested that the hybrid matrix with ZnO nanorod-based nanofiller could increase the permittivity of PVDF. Thereinafter, Shin *et al.*^22^ modified the previous structure by using ZnO nanoneedle instead of ZnO nanorod to improve the performance of hybrid structure. They showed that the incorporation of ZnO nanoneedle, which acted as nucleating agent, allowed for enhanced crystallinity, permittivity and reduced elastic modulus of the PVDF-based hybrid film. Choi *et al.*^23^ studied the origin of power enhancements in a hybrid piezoelectric structure comprised of ZnO NWs and PVDF polymer, compared to the electrical power from a pristine PVDF-based device.

Therefore, improved mechanical properties and piezoelectric response of hybrid structures has been reported. However, in all researches mentioned before, post-poling treatment is required to convert the nonpolar phase of PVDF to its polar phase which is a disadvantage of such hybrid structures due to complication of the required post-treatment process. Thus, it is required to modify the structure to have a more applicable hybrid piezoelectric device. For achieve this purpose, the electrospinning is a simple technique to obtain polar PVDF NFs directly from solution without need to post treatment.^24^ Thus, hybridization of PVDF nanofibers with ZnO nanorod, which has not been reported yet, may led to a novel hybrid device with modified structure.

Also, the mechanism of piezoelectric improvement of such hybrid piezoelectric device is rarely discussed. Thus, a qualitative study is needed to understand the mechanism of electrical enhancements in such novel hybrid nanogenerators.

Here, a novel hybrid piezoelectric structure based on electrospun PVDF NFs and vertically grown ZnO nanorods are developed. In our structure, electrospinning process allows to align the dipoles of PVDF without need to post electric poling. To investigate the effect of hybridization, the pristine PVDF NFs and pristine ZnO NRs nanogenerators were also fabricated.

The morphology and structure of PVDF NFs and ZnO nanorods were investigated. The piezoelectric response of three nanogenerators were measured under periodic impacts and were compared together. Mechanism of enhancement of electrical performance in our hybrid device were also discussed.

Development of versatile nanogenerators and sensors that use these hybrid and flexible structures can profoundly revolutionize the power harvesting and pressure sensing applications in smart clothing development.

## Results and discussion

**Fig. 1:**
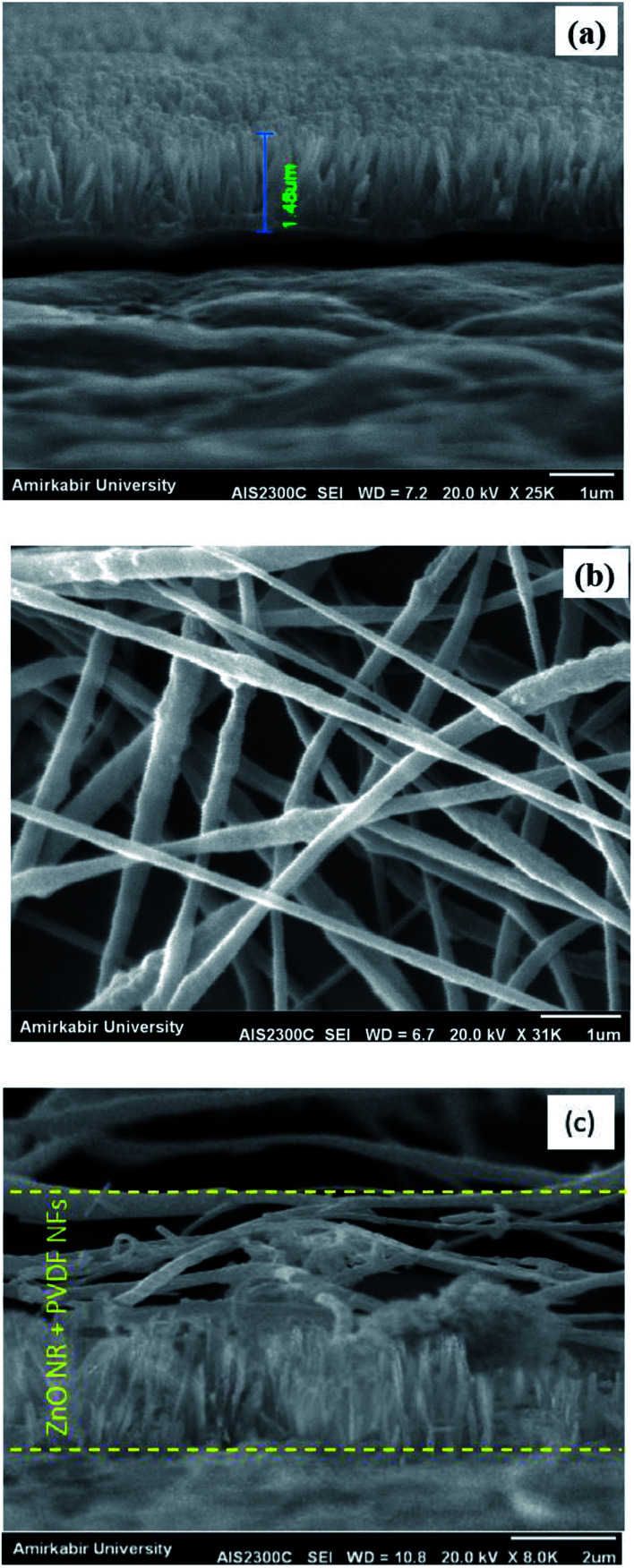
SEM image of (a) cross section of ZnO NRs grown on gold-coated PTFE (b) surface of electrospun PVDF NFs, (c) cross section of PVDF/ZnO.

Fig. 1(a–c) shows the SEM images of the synthesized ZnO nanorods, PVDF NFs and hybrid PVDF NFs/ZnO NRs.

The cross section view image of ZnO NRs (Fig. 1(a)) shows that uniform, high density and well aligned ZnO NRs are grown vertically along the *c*-axis direction on the PTFE substrate. The length and diameter of the nanorods are around 1.5 μm and 120 nm, respectively. Fig. 1(b) shows the SEM image of PVDF NFs mat surface. It is demonstrated that relatively uniform and randomly oriented fibers with the mean diameter of about 160 nm were fabricated. The cross section view of PVDF/ZnO hybrid was shown in Fig. 1(c).

**Fig. 2:**
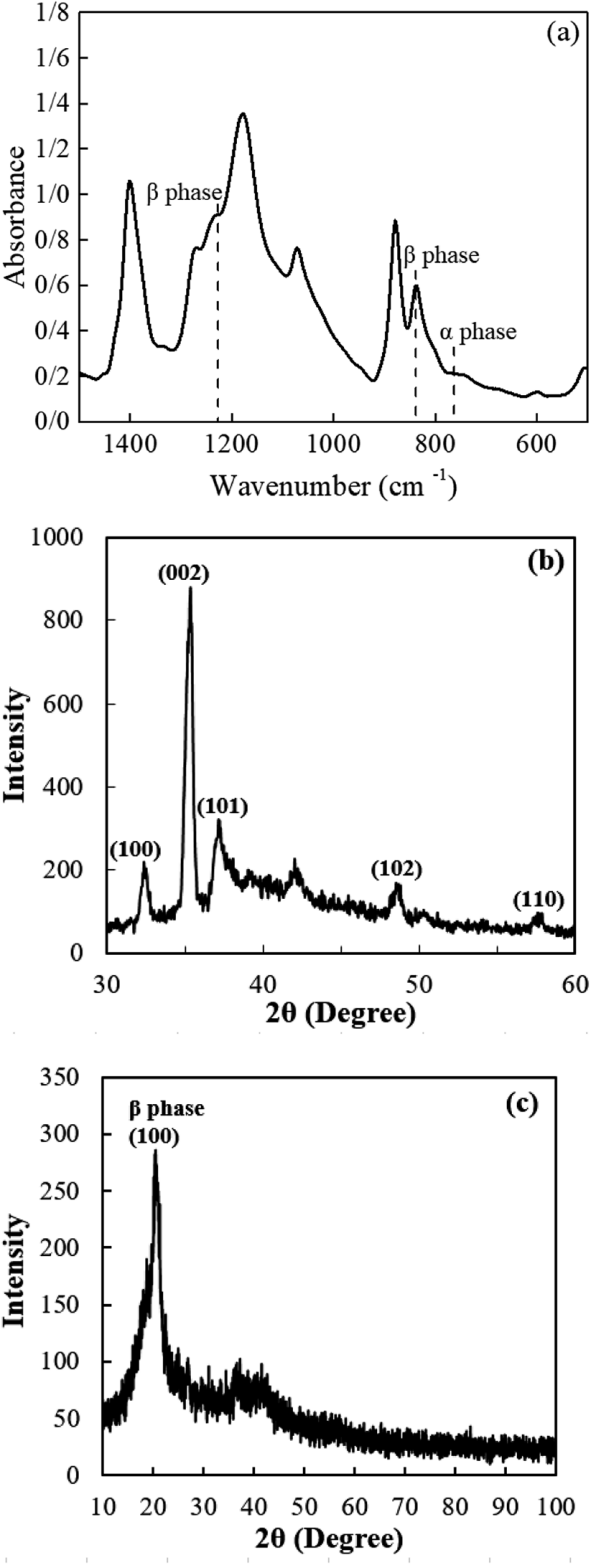
(a) FTIR spectra of electrospun PVDF NFs, (b) XRD pattern of ZnO NRs grown on PTFE, (c) XRD pattern of PVDF NFs.

Piezoelectric properties of PVDF are strongly depended on polar phases. FTIR spectroscopy is a proper technique to provide information about polar and non-polar phases in the structure of PVDF NFs. FTIR spectrum of PVDF NFs is shown in Fig. 2(a).

Except the small peak located at 763 cm^−1^ representing the characteristic bands of α phase, no other significant absorption bands related to α phase can be observed in the FTIR spectrum. The peaks at 840 cm^−1^ and 1232 cm^−1^ are related to β phase. As observed from the figure, PVDF NFs are predominantly in the polar phase. The FTIR spectroscopy (Fig. 2(a)) can be used to determine β phase proportion within the electrospun PVDF using the following equation:1
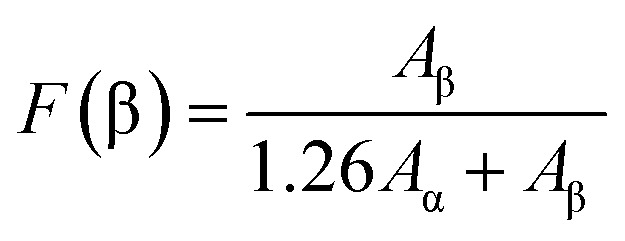
*A*_α_ and *A*_γ_ are the absorbance at 763 cm^−1^ (α phase) and 840 cm^−1^ (β phase). The calculated β phase content of PVDF NFs was 90%, indicating the direct formation of polar phase from the solution during the electrospinning process.^25^

X-ray diffraction analysis was performed to characterize ZnO NRs and PVDF NFs structures as shown in Fig. 2(b and c). In Fig. 2(b), the peaks at 2*θ* values of 32.44°, 35.29°, 37.23°, 42.16° and 48.58° are attributed to (100), (002), (101), (102) and (110) diffraction planes of the hexagonal structure of ZnO, respectively, with a lattice constant of *c* = 0.5206 nm.

The dominant peak related to the (002) diffraction plane demonstrates a high orientation degree along the *c*-axis direction perpendicular to the substrate.^26^ Both XRD and SEM confirm the formation of wurtzite hexagonal structure of the ZnO NRs.

Fig. 2(c) shows the crystal structure of PVDF NFs. The major peak at 20.5° corresponds to the diffraction from (100) plane related to polar β phase.^27^ No diffraction peak attributed to nonpolar α phase is observed in the XRD pattern of PVDF NFs, which is a clear evidence that β phase crystals were formed in electrospun PVDF mat. These results are consistent with FTIR spectroscopy data, relating to mechanical stretching and electric poling treatments during electrospinning process that induce the polar β phase without the need to any post-treatment. Also, polarized ZnO may also assist as nuclei to induced β phase of PVDF during the process.

**Fig. 3:**
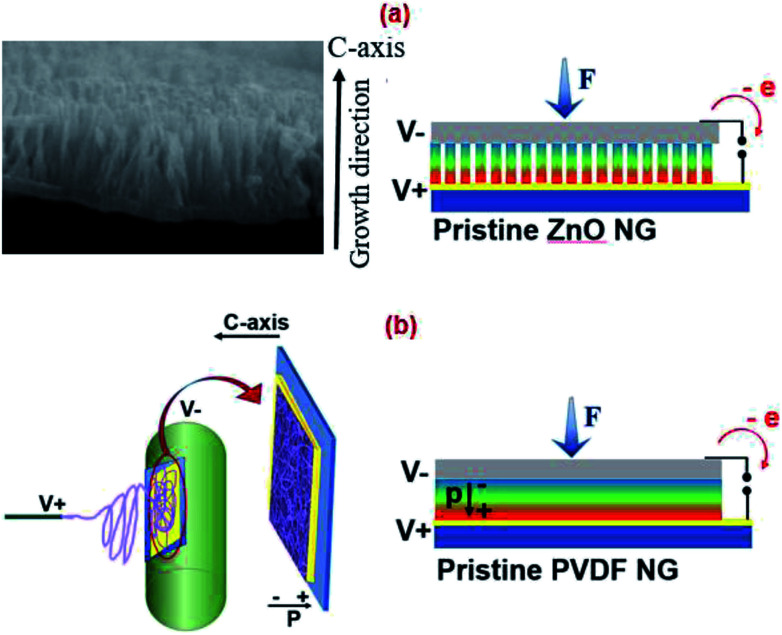
Schematic of proposed working mechanisms of (a) pristine ZnO NRs nanogenerator and (b) pristine PVDF NFs nanogenerator.

To explore the piezoelectric performance of prepared nanogenerators, the devices were subjected to periodic forces. By applying force on each of nanogenerator device, mechanical energy is converted into electricity by a scenario. The schematic of proposed working mechanism of pristine ZnO NRs and pristine PVDF NFs nanogenerators, are shown in Fig. 3(a and b).

In ZnO NRs, the power generation mechanism is the result of coupling of its semiconductor and piezoelectric properties which leads to the creation of a strain field and charge separation across the rods when an external strain is applied to rods.^28^ In PVDF, piezoelectricity relies on its polar crystalline phase that has a net non-zero dipole moment, leading to the generation of piezopotential in response to an applied external force.

In a hybrid structure, piezopotential is generated from two piezoelectric components; PVDF and ZnO NRs. The polar direction of ZnO NRs is along *c* axis. When a compressive force is applied on the top surface of nanowires along *c*-axis (growth direction), this generates a piezoelectric field inside the ZnO that separates electrical charges and drives electrons flow from an Au electrode to the upper side of nanorods so that the top side has a negative charge and the bottom end has a positive charge.^29^ Also in a PVDF electrospun mat, dipoles are along the *c* axis (mat thickness direction) owing to orientation of the CF_2_ dipoles due to stretching and high electric field applied during electrospinning.^30^

Therefore, in the hybrid structure, the piezopotential drop generated from two piezoelectric components can be added positively, because they have the same polarity. Aligning of dipoles is an important issue to hybridize PVDF and ZnO constructively, resulting the electrical enhancement of hybrid structure compared to pristine ones. The generated piezopotential distribution introduces induced charges in the top and bottom electrodes, and consequently generates the output voltage.

Since the aim of this work is toward self-powered devices and wearable energy harvesters capable of harvesting mechanical energy from human activity, piezoelectric output of nanogenerators were evaluated under low frequency periodic deformations. Two types of deformation include vertical impacts and bending motion were applied to nanogenerators.

**Fig. 4:**
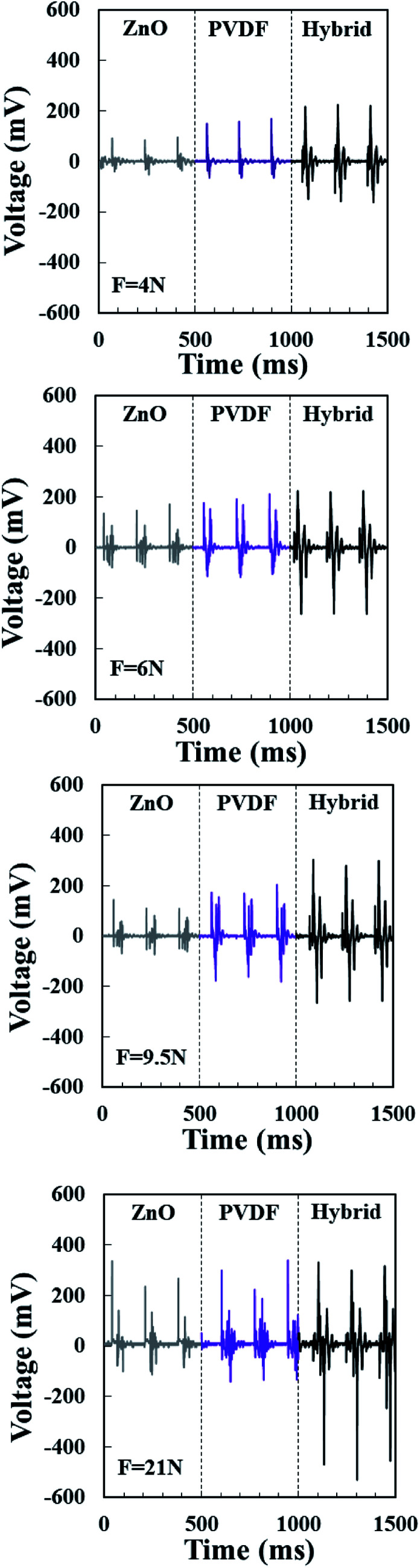
Output voltage of pristine PVDF NFs, pristine ZnO NRs and PVDF NFs/ZnO NRs hybrid nanogenerator under periodic impacts with forces of 4, 6.5, 9 and 21 N at the frequency of 6 Hz.

The cyclic vertical impacts are similar to the human walking/running motion. Hence, the nanogenerator can be used in shoes pad. Piezoelectric response of nanogenerators under vertical impacts was recorded by a customized impact testing platform. The nanogenerators were mounted between two plates installed on impact stage, by which periodic forces with a varied frequency were applied on the NGs. The device's wires directly connected to the oscilloscope to measure the piezoelectric performance of nanogenerators. The same measurement process was carried out on all nanogenerators for comparison. Fig. 4 shows the output voltage of pristine PVDF NFs, pristine ZnO NRs and PVDF NFs/ZnO NRs hybrid nanogenerators under the periodic mechanical forces of 4, 6.5, 9 and 21 N. The frequency in all tests was 6 Hz.

To explore the effect of hybridization on piezoelectric performance, the output voltage of hybrid devices and pristine devices at a same force were compared. The peak to peak voltage (*V*_p–p_) value of nanogenerator devices can be extracted from Fig. 4. At *F* = 4 N, the *V*_p–p_ of pristine ZnO and pristine PVDF are 128 and 216 mV, respectively. This value increases to 356 mV for the hybrid structure at the same force that is 2.8 times larger than pristine ZnO and 1.6 times larger than pristine PVDF. It can be clearly seen, in all forces, the piezoelectric output of the hybrid structure is larger than both the pristine PVDF and the pristine ZnO nanogenerators.

At *F* = 21 N, the *V*_p–p_ of hybrid nanogenerator is 2 times larger than both pristine ones. This increase is owing to the similarity of poling direction of PVDF NFs and ZnO NRs so that their piezoelectric effect can be constructively added when a force applied on the device. Also, it can be seen that the piezoelectric response of ZnO NRs, at small forces, is smaller than pristine PVDF. This can be explained based on the number of nanorods contributed in final output. In small forces, since all nanorods are not exactly at the same height, some nanorods may not be in contact with the electrode or may not be deformed under force. While, at *F* = 21 N, the output voltage of pristine PVDF and pristine ZnO are almost the same. These results can be explained based on an increase in the number of nanorods contacting the electrode by increasing the applied force. To investigate the response of devices in terms of applied force, we compared the *V*_p–p_ of nanogenerators under different forces. As shown in Fig. 4, for all devices, the voltage outputs increase with the forces applied, indicating the dependence of piezoelectric response to the external forces.

To measure the output current, the nanogenerator devices were connected to a charge amplifier circuit. The output of the circuit was sent to an oscilloscope, indicating the electrical output charge.

**Fig. 5:**
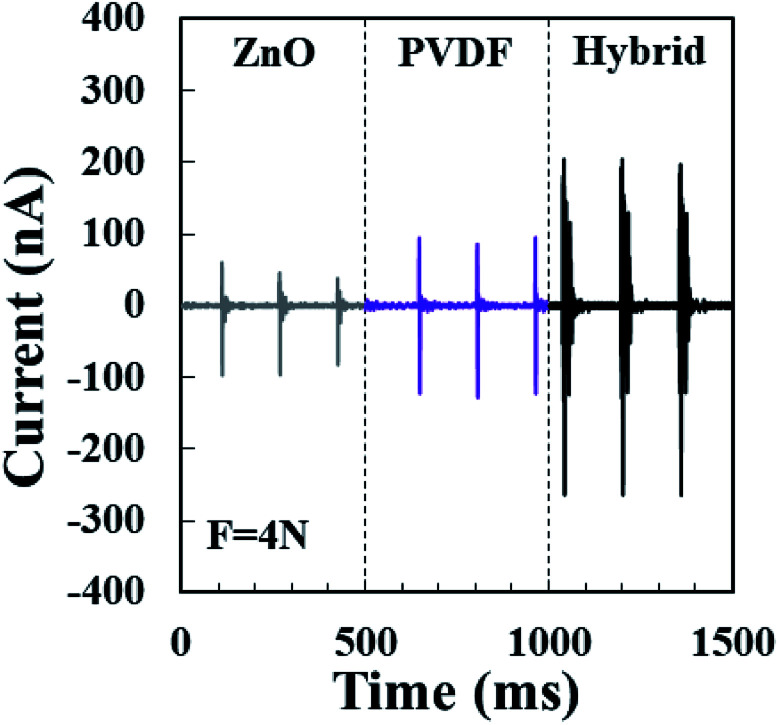
Output current of pristine PVDF NFs, pristine ZnO NRs and PVDF NFs/ZnO NRs hybrid nanogenerator at *F* = 4 N and frequency of 6 Hz.

The output current was extracted from the output charge by 
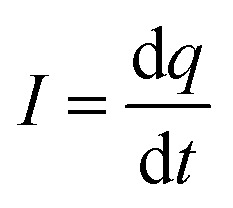
. The output current of pristine PVDF NFs, pristine ZnO NRs and PVDF NFs/ZnO NRs hybrid nanogenerator were measured at *F* = 4 N with the frequency of 6 Hz. The results are shown in Fig. 5. The measured output currents for pristine ZnO NRs and pristine PVDF are 114 nA and 212 nA, respectively. This value reaches 456 nA for the hybrid structure under the same condition. It means that the current output increases up to 4 times larger than the pristine ZnO and 2 times larger than the PVDF devices. This value reaches 456 nA for the hybrid structure under the same condition. It means that the current output increases up to 4 times larger than the pristine ZnO and 2 times larger than the PVDF devices. Overall, the output voltage and output current results showed that the use of two piezoelectric components in a hybrid structure nanogenerator results in a significant improvement of the generated power up to 11 times larger than the nanogenerators based on pristine ZnO and 3.2 times larger than the pristine PVDF at *F* = 4 N.

**Fig. 6:**
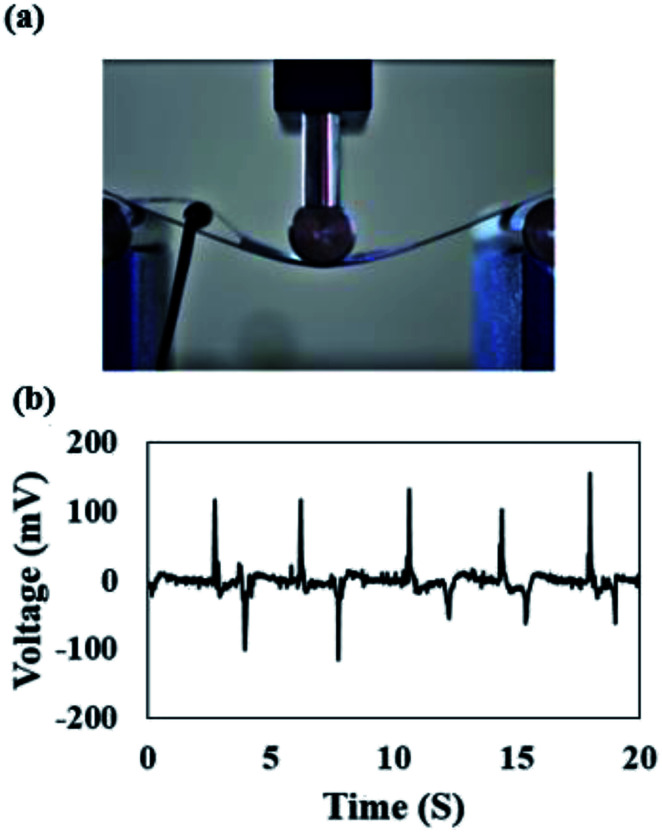
(a) View of hybrid nanogenerator under three-points bending test, (b) output voltage of hybrid nanogenerator under periodic bending deformation.

Piezoelectric response of the nanogenerators were also measured under bending deformation at low frequency, simulating the body motion such as folding and releasing action of the elbow and knee joints. As Fig. 6(a) demonstrates, the three-points bending test was carried out on the specimen to apply the external strain. The specimen voltage–time response is also given in Fig. 6(b). This results indicate that our hybrid structure suggests a highly promising nanogenerator to scavenge energy from the human activity.

## Experimental section

### Preparation of PVDF solutions

PVDF pellets (*M*_w_ ∼ 530 000), dimethylformamide (DMF) and acetone were purchased from Sigma Aldrich. The PVDF solution was prepared by adding PVDF pellets with the concentration of 18% to DMF/acetone (60/40) and stirring for 3 h at 60 °C to obtain a homogeneous and transparent solution.

### Growth of ZnO NRs

First, a rectangular region on the surfaces of PTFE flexible substrate (200 μm in thickness) was coated with a gold layer (5 nm in thickness) by sputtering. Then, 0.005 M zinc acetate dihydrate (98%, Aldrich) in ethanol was spin-coated over the gold-coated PTFE film. This layer was rinsed with ethanol and then dried at room temperature. To have a full coverage of substrate with zinc acetate, the coating was performed three times. Then the substrate was heated to 90 °C for 30 min. The deposition and decomposition steps were repeated twice to ensure that a uniform seed layer was formed to cover the substrate.^31^ A hydrothermal method was used to grow ZnO nanorods on the seed layer. The prepared seed layer was kept in an aqueous solution of zinc nitrate hexahydrate [Zn(NO_3_)_2_·6H_2_O, Sigma-Aldrich, 0.01 M] and hexamethylenetetramine (HMT) [C_6_H_12_N_4_, Sigma-Aldrich, 0.01 M] at 90 °C for 3 h. Finally, the resultant layer containing the ZnO nanorods was rinsed with DI water and dried at room temperature.

### Fabrication of nanogenerators

To fabricate the hybrid device, the prepared ZnO NRs grown on gold-coated PTFE was pasted on the rotating collector of electrospinning instrument, in which the PVDF NFs were electrospun at the rate of 0.5 ml h^−1^ on it. The syringe tip to collector distance was fixed at 15 cm and the applied voltage was 15 kV. The speed of collector was fixed at 500 rpm. The electrospinning continued until ZnO NRs were fully covered with PVDF NFs. Finally, a conductive Al foil, as the top electrode, was pasted on electrospun PVDF NFs by silver paste. The Au layer, which coated on PTFE, were served as the bottom electrode. To complete the device, Cu wires were connected to the top and bottom electrodes.

**Fig. 7:**
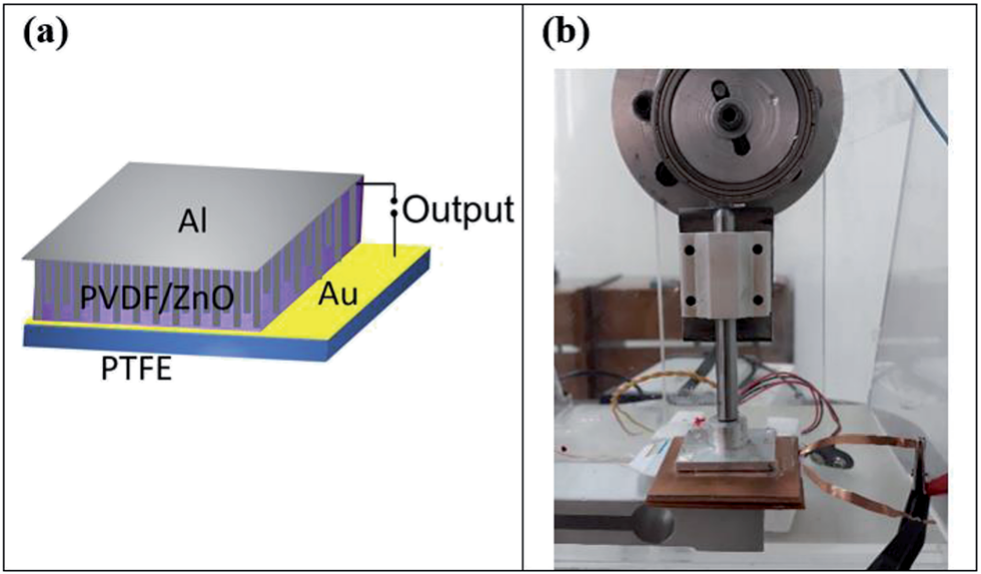
(a) Schematic of the PVDF/ZnO hybrid nanogenerator devices, (b) experimental setup to measure output voltage/current of nanogenerator devices.

To explore the effect of hybridization on piezoelectric output, the pristine ZnO NRs nanogenerator and pristine PVDF NFs nanogenerator were also fabricated. To prepare the pristine PVDF NFs nanogenerator, PVDF NFs were electrospun on a gold-coated PTFE in the same way as that explained before, then an Al foil was pasted on it by silver paste as the top electrode. The pristine ZnO NRs nanogenerator was fabricated by pasting an Al foil directly on the surface of ZnO NRs grown on the gold-coated PTFE. The effective area of all nanogenerators was 3 cm^2^ (1.5 cm × 2 cm). The schematic of prepared hybrid nanogenerator devices is shown in Fig. 7(a).

### Characterization and measurements

The surface morphology of the ZnO NRs and PVDF NFs, were investigated by a scanning electron microscope (SEM, XL-30E Philips Co., Holland). Cross section images of ZnO NRs were taken by a SEM (SEM (AIS2100, Seron technologies, South Korea)). PTFE layers were coated with gold by a sputter coater (Bal-Tec, Germany). To analyze the crystal structures of ZnO NRs and PVDF NFs, a X-ray diffraction (Equinox 3000model, INEL France Co.) equipped with CuKα radiation (*λ* = 0.1564 nm) was used and operated at 30 V and 15 mA in the 2*θ* range of 30–60° at the scanning speed of 1.8° min^−1^. To characterize the β phase content of PVDF NFs, FTIR spectroscopy (PerkinElmer Spectrum 400 spectrophotometer) measurement was carried out in the range of 400–4000 cm^−1^ with the resolution of 2 cm^−1^. The piezoelectric output of the nanogenerators was measured by applying two types of periodic deformation consist of vertical impact and bending deformation. The employed impact experimental setup consists of an impact test rig, a load cell and an oscilloscope to monitor the output response. To measure the output charge, in addition to above mentioned instruments, a charge preamplifier was also used. Then, by using 
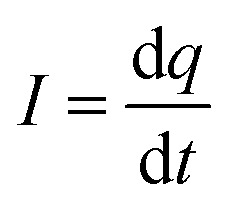
, the output current of the devices was calculated from the output charge. Additionally, three-points bending test was performed to apply periodic bending deformation to the hybrid nanogenerator at the low frequency. The view of the impact setup is shown in Fig. 7(b).

## Conclusions

We reported the electrical performance and the mechanism of electrical enhancement in a novel hybrid piezoelectric structure based on electrospun PVDF NFs and vertically grown ZnO nanorods. The hydrothermal method was used to grow vertical nanorods on a gold coated PTFE. The electrospinning was also used to prepare PVDF NFs with high percentage of polar phase, with no need to electrical poling. The PVDF nanofibers were electrospun on those ZnO NRs already grown on PTFE to fabricate a hybrid structure. The morphology and structure of ZnO NRs and PVDF NFs were investigated by SEM, XRD and FTIR. To measure the piezoelectric output of the hybrid nanogenerator, the prepared devices were placed under periodic impacts. To investigate the effect of hybridization of two piezoelectric materials on the output, the pristine ZnO NRs nanogenerator and pristine PVDF NFs nanogenerator devices were also fabricated and their output was measured under same conditions. The electrical output power of our hybrid NG was found to be enhanced compared to that from the NG based on pristine ZnO and pristine PVDF, respectively. The origin of the power enhancement of the hybrid structure is mainly due to synergic piezoelectric properties of two piezoelectric materials resulted from same poling direction in ZnO and PVDF in the hybrid structure. This simple hybrid device is a promising nanogenerator to convert the mechanical movements, especially human motions, more efficiently into electricity for actual applications.

## Conflicts of interest

There are no conflicts to declare.

## Supplementary Material

RA-009-C8RA10315A-s001

RA-009-C8RA10315A-s002
